# Transjugular intrahepatic portosystemic shunt, local thrombaspiration, and lysis for management of fulminant portomesenteric thrombosis and atraumatic splenic rupture due to vector-vaccine-induced thrombotic thrombocytopenia: a case report

**DOI:** 10.1186/s13256-022-03464-x

**Published:** 2022-07-11

**Authors:** Sandra Emily Stoll, Patrick Werner, Wolfgang A. Wetsch, Fabian Dusse, Alexander C. Bunck, Matthias Kochanek, Felix Popp, Thomas Schmidt, Christiane Bruns, Bernd W. Böttiger

**Affiliations:** 1 Department of Anesthesiology and Intensive Care Medicine, Faculty of Medicine and University Hospital of Cologne, University of Cologne, Cologne, Germany; 2 Department of Diagnostic and Interventional Radiology, Faculty of Medicine and University Hospital of Cologne, University of Cologne, Cologne, Germany; 3 Department I of Internal Medicine, Intensive Care Medicine, Faculty of Medicine and University Hospital of Cologne, University of Cologne, Cologne, Germany; 4 Department of General, Visceral, Tumor and Transplantation Surgery, Faculty of Medicine and University Hospital of Cologne, University of Cologne, Cologne, Germany

**Keywords:** VITT, VIPIT, ChAdOx1 nCoV-19 vaccination, COVID vaccination, TIPS

## Abstract

**Introduction:**

Recombinant adenoviral vector vaccines against severe acute respiratory syndrome coronavirus 2 have been observed to be associated with vaccine-induced immune thrombotic thrombocytopenia. Though vaccine-induced immune thrombotic thrombocytopenia is a rare complication after vaccination with recombinant adenoviral vector vaccines, it can lead to severe complications. In vaccine-induced immune thrombotic thrombocytopenia, the vector vaccine induces heparin-independent production of platelet factor 4 autoantibodies, resulting in platelet activation and aggregation. Therefore, patients suffering from vaccine-induced immune thrombotic thrombocytopenia particularly present with signs of arterial or venous thrombosis, often at atypical sites, but also signs of bleeding due to disseminated intravascular coagulation and severe thrombocytopenia. We describe herein a rare case of fulminant portomesenteric thrombosis and atraumatic splenic rupture due to vaccine-induced immune thrombotic thrombocytopenia.

**Case summary (main symptoms and therapeutic interventions):**

This case report presents the diagnosis and treatment of a healthy 29-year-old male Caucasian patient suffering from an extended portomesenteric thrombosis associated with atraumatic splenic rupture due to vaccine-induced immune thrombotic thrombocytopenia after the first dose of an adenoviral vector vaccine against severe acute respiratory syndrome coronavirus 2 [ChAdOx1 nCoV-19 (AZD1222)]. Therapeutic management of vaccine-induced immune thrombotic thrombocytopenia initially focused on systemic anticoagulation avoiding heparin and the application of steroids and intravenous immune globulins as per the recommendations of international societies of hematology and hemostaseology. Owing to the atraumatic splenic rupture and extended portomesenteric thrombosis, successful management of this case required splenectomy with additional placement of a transjugular intrahepatic portosystemic shunt to perform local thrombaspiration, plus repeated local lysis to reconstitute hepatopetal blood flow.

**Conclusion:**

The complexity and wide spectrum of the clinical picture in patients suffering from vaccine-induced immune thrombotic thrombocytopenia demand an early interdisciplinary diagnostic and therapeutic approach. Severe cases of portomesenteric thrombosis in vaccine-induced immune thrombotic thrombocytopenia, refractory to conservative management, may require additional placement of a transjugular intrahepatic portosystemic shunt, thrombaspiration, thrombolysis, and surgical intervention for effective management.

## Introduction

SARS-CoV-2-vaccine-induced immune thrombotic thrombocytopenia (VITT) represents a rare complication after vaccination with adenoviral vector vaccines, with incidence from 1 case in 125,000 to 1 case in 1 million [1, 2]. So far, most such patients have presented with signs of arterial or venous thrombosis at atypical sites, but disseminated intravascular coagulation (DIC) has also been described to be associated with vector vaccines [1–9]. It is assumed that VITT is caused by the production of autoantibodies against the complex of platelet factor 4 (PF4) and an as-yet-unknown polyanion found in recombinant adenovirus vaccine [10]. VITT occurs around 4–28 days post vaccination, and frequently this disease takes a fatal course [3, 11, 12]. This report describes a severe case of VITT complicated by extended portomesenteric thrombosis associated with atraumatic splenic rupture, an extremely rare complication of VITT. To the best of the authors’ knowledge, this is the first description of such a case involving therapeutic anticoagulation, treatment of hemorrhagic coagulopathy, application of intravenous immune globulins, splenectomy, TIPS placement, thrombaspiration, and thrombolysis for effective management of VITT.

## Case presentation

### Patient information and initial clinical findings

We report the case of a healthy 29-year-old Caucasian, married, male patient with no major past medical history except Vitiligo’s disease presenting with signs of vector-vaccine-induced thrombotic thrombocytopenia (VITT) 11 days post vaccination with an adenoviral vector vaccine against SARS-CoV-2 [ChAdOx1 nCoV-19 (AZD1222)]. The patient had no history of exposure to tobacco, alcohol, or drugs and presented with an unremarkable family, environmental, employment, and psychosocial history. He exercised regularly and was on a vegetarian diet. The patient did not take any chronic medication and had no known allergies. He presented 11 days post vaccination to the outpatient emergency medical service with nausea and abdominal pain progressing for the previous 2 days. On physical examination the patient was fully orientated [Glasgow Coma Scale (GCS) 15] without any neurological pathologies and showed stable vital parameters (respiratory rate 15/minute, SpO_2_ 97%, heart rate 80/minute, blood pressure 123/62 mmHg, body temperature 36.7 °C). He was consecutively discharged, being suspected of suffering from food poisoning. However, the patient’s condition deteriorated, resulting in hospital admission the next day with symptoms of vomiting, hemoptysis, melena, fever, and deteriorating health condition. His blood pressure was 90/60 mmHg, his heart rate 100/minute, his respiratory rate 25/minute, his SpO_2_ 97%, and his body temperature was 36.9 °C. Presenting with a GCS of 15, there were no pathological findings on neurological examination. The patient’s full blood count on admission showed severe thrombocytopenia (13,000/µl).

### Initial clinical management

**Fig. 1:**
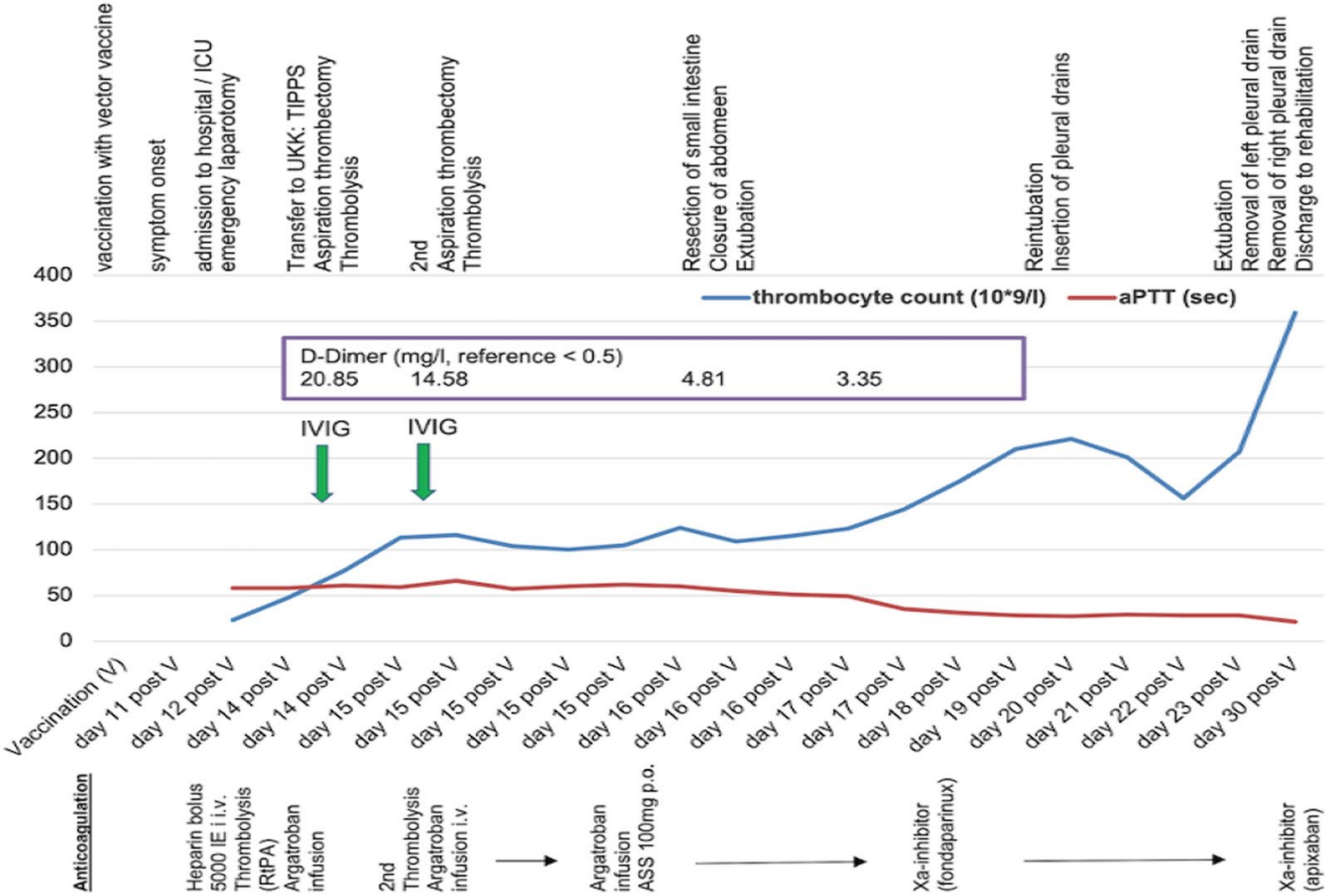
Timeline of VITT

**Fig. 2:**
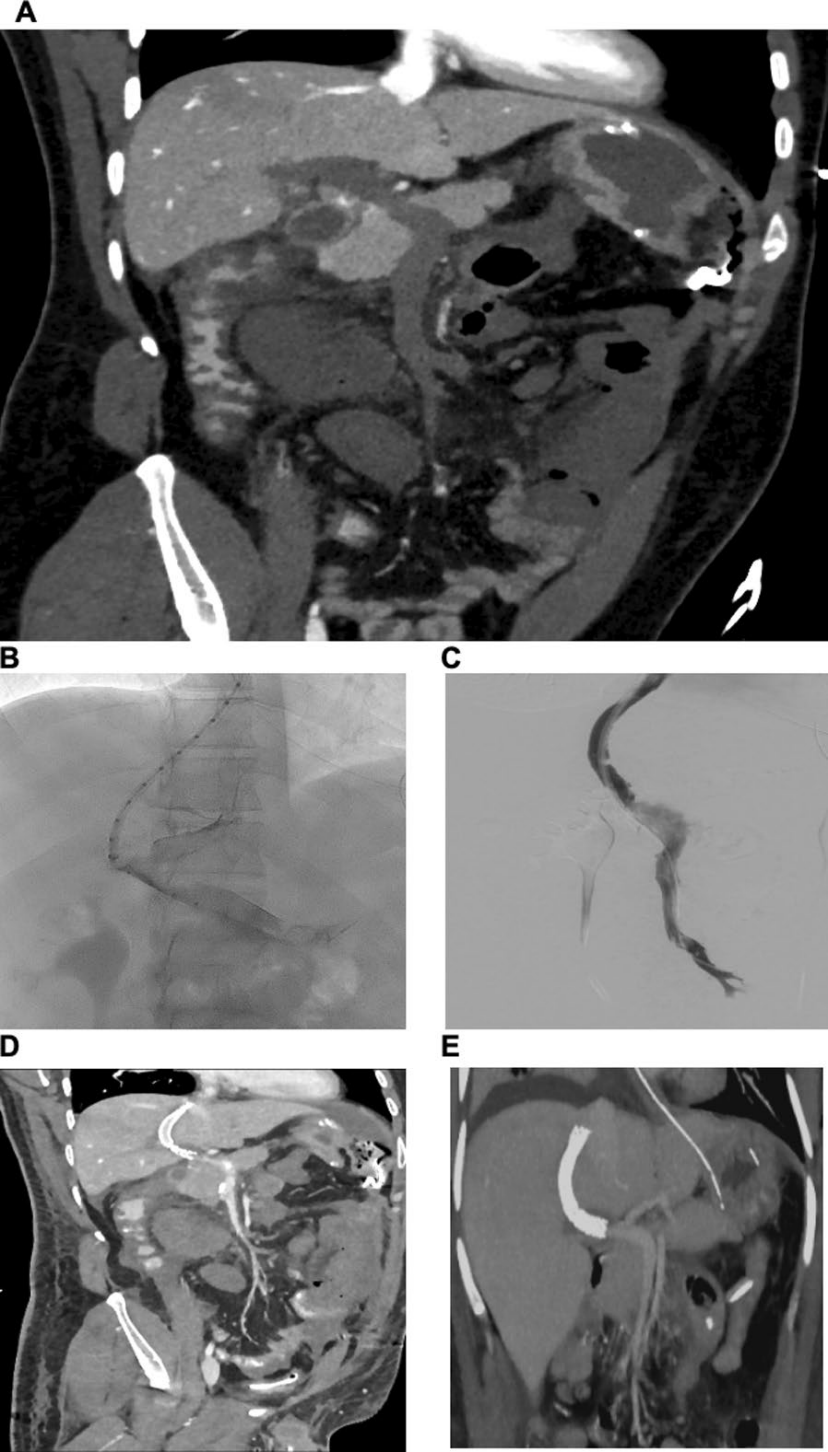
Radiographic images of portomesenteric thrombosis and TIPS insertion. **A** Coronal computed tomography image, showing extended portomesenteric thrombosis pre-TIPS. **B** Angiography showing portal thrombosis and insertion of measuring catheter. **C** Angiography showing TIPS insertion, mild residual thrombosis after thrombaspiration, and local lysis. **D** Coronal computed tomography (CT) post TIPS and local lysis. **E** Coronal computed tomography (CT) post TIPS CT and post resection of small intestine, restituted portomesenteric flow with no signs of relevant residual thrombosis

The patient was transferred to the intensive care unit (ICU). An abdominal computed tomography (CT) showed free perihepatic, perisplenic fluid and active endoluminal bleeding at the ileocecal passage (spot sign). An esophagogastroduodenoscopy and colonoscopy were performed, detecting a congestive gastropathy and hemorrhagic gastritis with no signs of active bleeding. The patient received an intravenous proton pump inhibitor (40 mg pantoprazole intravenously). Anemia and thrombocytopenia were treated by transfusion of 4 units of red blood cells (RBC) and 2 units of platelets (PLT). Due to the lack of the past medical history of the patient regarding his vaccination, autoimmune thrombocytopenia was suspected, and a dose of intravenous corticosteroids was given (500 mg prednisolone). The next day, the patient developed hemorrhagic shock. The patient was intubated and sedated with intravenous propofol 2% (2 mg/kg/hour) and sufentanil (30 µg/hour) infusion and required continuous infusion of noradrenaline (0.2 µg/kg/minute) to stabilize his blood pressure. Spontaneous atraumatic splenic rupture, diagnosed on abdominal ultrasound, required emergent exploratory laparotomy and splenectomy. The small intestine presented with ischemic demarcation. Due to extended coagulopathy, intraoperative blood loss of 3 liters required massive transfusion of 8 units of RBC, 2 lyophilized units of plasma, 2 units of PLT, 1 g tranexamic acid, 2 g fibrinogen, and 1800 units of prothrombin complex. The abdomen was packed and left open. With persisting hemodynamic instability and hemorrhagic coagulopathy (see laboratory values in Table 1), the patient was transferred (intubated and sedated) to our tertiary hospital ICU (University Hospital of Cologne, UKK; see timeline of VITT in Fig. 1). Another abdominal CT detected dilated small intestine with edematous thickened walls and occlusion of the portal, splenic, and superior mesenteric vein (Fig. 2A). Colleagues from the Department of General Surgery and the Department of Radiology decided conclusively due to the extended thrombosis to firstly reduce the venous congestion and restore portomesenteric blood flow by thrombectomy and placement of a TIPS: A 10 F sheath was inserted via the right jugular vein, and 5000 units of unfractionated heparin were injected intravenously. A thrombectomy catheter was inserted via the TIPS into the superior mesenteric vein (Fig. 2B, C), and an interventional aspiration thrombectomy and local lysis therapy was initiated: a bolus of 10 mg tissue-type plasminogen activator (rtPA, Alteplase) followed by continuous intravenous infusion of rtPA (at a rate of 1.5 mg/hour). During the procedure, there was another blood loss of approximately 1 liter, requiring the transfusion of a further 3 units of RBC and 2 units of PTL. Blood pressure was stabilized with continuous intravenous noradrenaline (0.4 µg/kg/minute) and vasopressin (2 Units/hour) infusion. The intravenous dose of pantoprazole was increased to 160 mg (continuous infusion) per day for the next 3 days. Due to the suspicion of ventilator-associated pneumonia, treatment with intravenous piperacillin/tazobactam was initiated (3 × 4.5 g/day) for 7 days of treatment. Microbiology screening of tracheal secretions revealed small amounts of *Serratia liquefaciens* (sensitive to piperacillin/tazobactam) and *Candida albicans*. Under antimicrobial treatment, inflammatory markers decreased and respiratory function improved.

**Table 1:** Laboratory values on admission to intensive care unit (ICU), University Hospital Cologne (UKK)

Test	Result	Normal range
Hemoglobin (g/dl)	11.2	13.5–18
Thrombocytes (/µl)	23000	150,000–400,000
Fibrinogen per Clauss (g/l)	1.2	2.1–4
D-Dimer (mg/l)	20.6	< 0.5
ALAT (U/l)	14	< 50
ASAT (U/l)	23	< 50
γ-GT (U/l)	10	< 60
Bilirubin total (mg/dl)	6	< 1.2
Creatinine (mg/dl)	0.88	0.5–1.1
Potassium (mmol/l)	4.5	3.5–4.5
Sodium (mmol/l)	136	135–135
Lactate (mmol/l)	6	0.5–2.2
Quick value (%)	78	70–120
INR	1.1	< 2
aPTT (s)	58	< 36

*ALAT* Alanine transaminase; *ASAT* Aspartate aminotransferase; *γ-GT* γ-glutamyl transferase; *INR* International Normalized Ratio; *aPTT* Activated partial thromboplastin time

### Timeline

#### Suspicion of VITT: diagnostic assessment and therapeutic intervention

Parallel to this intervention, the patient’s past medical history revealed that he had received his first vaccination dose of a vector vaccine (ChAdOx1 nCoV-19 (AZD1222), AstraZeneca) 14 days before. This finally raised suspicion of VITT, and intravenous immune globulins (IVIG) were administered to the patient: 80 g IVIG (1g/kg body weight Octagam®; Octapharma GmbH, Langenfeld, Germany, for the patient’s body weight of 80 kg) for two consecutive days. Before administration of IVIG, blood samples were taken for immunologic assays for heparin-induced thrombocytopenia (HIT) type II, thrombocytopenia, and thrombophilia screening (Table 2). Anticoagulation was managed with a direct thrombin inhibitor (argatroban with dosage of 0.15–0.25 µg/kg/minute). Coagulation screening [blood count, plasmatic coagulation, thrombelastometry (ROTEM)] was run 6-hourly. A rapid HIT II screening test (ID-PaGIA heparin/PF4-antibody test kit, Bio-Rad Laboratories GmbH, Feldkirchen, Germany) showed a negative result. An IgG-specific enzyme-linked immunosorbent assay (ELISA) (Immunocor GTI Diagnostics, Inc., Waukesha, WI USA) was positive (odds ratio 2.35). With hemodynamic stabilization of the patient, continuous intravenous infusion of vasopressin could be stopped and parenteral feeding initiated.

**Table 2:** Thrombophilia and thrombocytopenia screening

Test	Result	Normal range
aPTT (lupus sensitive)	25.2 seconds	25.1–36.5 seconds
Lupus anticoagulant (dRVVT)	Negative	–
Cardiolipin antibodies (IgG, IgM)	Negative	–
Antiphospholipid antibodies	Negative	–
β2-Glycoprotein antibodies (IgG, IgM)	Negative	–
Homocysteine	13.6 μmol/l	0–15 μmol/l
Factor VIII	120%	50–150%
APC resistance	Negative	–
Protein C activity	53%	70–140%
Factor V Leiden mutation (G1691G wild type)	Negative	–
Prothrombin mutation (G20210A wild type)	Negative	–
Haptoglobin	232 mg/dl	20–200 mg/dl
Paroxysmal nocturnal hemoglobinuria	Negative	–
ADAMTS-13 activity	67%	50–150%
C3 complement (g/l)	0.28	0.9–1.8
C4 complement (g/l)	0.05	0.1–0.4
Fragmentocytes	< 1%	< 1%
Rapid HIT assay	Negative	–
IgG-specific ELISA (PF4 antibodies)	Positive	–
HIPA (PF4/heparin; HIT type II)	Negative	–
PIPA (PF4-induced platelet aggregation/VITT)	Positive	–

*aPTT* Activated partial thromboplastin time; *dRVVT* Dilute Russell's viper venom time; *IgG* Immunoglobulin G; *IgM* Immunogobulin M; *APC-resistance* Activated protein C resistance; *ADAMTS-13* A disintegrin and metalloproteinase with a thrombospondin type 1 motif, member 13; *C3* Complement component; *C4* Complement component 4; *HIT* Heparin induced thrombocytopenia; *ELISA* Enzyme-linked immunosorbent assay; *PF4* Platelet factor 4; *HIPA* Heparin-induced platelet aggregation assay; *PIPA* Platelet factor 4-induced platelet activation; *VITT* Vaccine-induced immune thrombotic thrombocytopenia

Day 15 post vaccination (see timeline in Fig. 1), another angiography revealed reduced flow in the portal and superior mesenteric vein due to residual thrombus material. A further aspiration thrombectomy was performed, resulting in restoration of hepatopetal portomesenteric blood flow.

On day 16 post vaccination, the abdominal packing was removed, the ischemically demarked parts of the small intestine (190 cm) were resected, and the remaining intestine (approximately 100 cm) was connected in the form of a side-to-side duodeno-ileostomy. As the thrombocyte count continued to stabilize, thrombocyte aggregation could be blocked with acetylsalicylic acid (ASS 100 mg intravenously) to prevent clotting of the essential TIPS. The patient could be extubated, his abdominal pain was sufficiently treated with slow-release oral morphine (3 × 10 mg), and enteral feeding could be started.

On day 27 post vaccination, on the patient’s discharge to the general ward, the thrombocyte count had fully recovered to the reference range. Anticoagulation with the direct thrombin inhibitor (argatroban) was stopped, and treatment with fondaparinux was initiated. On day 31 post vaccination, with the patient’s discharge to the rehabilitation center, fondaparinux was replaced with an oral Xa-inhibitor (apixaban) for at least another 6 months. Due to the TIPS, the patient will remain on lifelong aspirin therapy.

### Follow-up and outcomes

The patient remained under monthly immunologic and hemostaseologic surveillance (screening of autoantibodies against PF4 and coagulation parameters). Immunologic analysis (Reference Laboratory for VITT, University Hospital Greifswald, Institute of Immunology and Transfusion Medicine, Germany, Prof. Greinacher) on the day of admission to UKK confirmed the diagnosis of VITT: HIPA test (heparin-induced platelet activation) was negative and PF4-induced platelet activation (PIPA) test (modified HIPA test) was positive. He remains anticoagulated with apixaban for another 6 months, plus lifelong therapy with acetylsalicylic acid for his TIPS. By following a 4-week rehabilitation program including physiotherapy and nutritional advice in the rehabilitation clinic, the patient fully recovered to a Barthel index of 100 out of 100 points and regained mental stability with no signs of anxiety or depression. After rehabilitation, he could return to full-time work at his former job as a purchasing agent for a public broadcasting institution. His socioeconomic situation has not changed due to the disease.

## Discussion

To the best of the authors’ knowledge, this report presents the first case of VITT complicated by extended portomesenteric thrombosis and resulting in atraumatic splenic rupture, which made splenectomy as well as TIPS placement inevitable. This led to a challenging combination: On the one hand, bleeding complications due to hemorrhagic coagulopathy because of splenic rupture and vaccine-induced thrombopenia required administration of clotting agents. On the other, extended portomesenteric thrombosis and the risk of TIPS occlusion necessitated therapeutic anticoagulation and platelet inhibition. This was managed by a broad interdisciplinary approach, close intensive care monitoring, and effective coordination of all treatment measures: application of intravenous immune globulins, adequate anticoagulation in VITT, as well as administration of clotting agents for the treatment of hemorrhagic coagulopathy, an emergency splenectomy, and placement of a TIPS for thrombaspiration and thrombolysis.

### Symptoms and risk factors of VITT

Most cases of VITT documented in literature presented clinically with the triad of significantly elevated D-dimers (up to five times the reference range), thrombocytopenia < 150,000/µl, and arterial and/or venous thrombosis (especially at unusual sites), as did our patient. Portomesenteric thrombosis has been described in VITT previously, but not as fulminant as presented in our case with atraumatic splenic rupture requiring TIPS insertion and emergent splenectomy. The triad of symptoms in VITT resembles HIT type II and the more rare spontaneous HIT without heparin exposure [13]. As per the recommendations of international professional societies [14–16], clinical trials of VITT presenting 4–48 days (median, 14) [11] post vaccination, mostly after the first dose of vector vaccine, should raise suspicion of VITT and must be investigated accordingly [12]. So far, there are no identifiable risk factors for VITT except the vaccination with recombinant adenoviral vector vaccines itself, with an age range of VITT from 18 to 79 years (median 48 years) and equal gender distribution [17].

### Pathophysiology of VITT

Vaccination with recombinant adenoviral vector vaccines can result in heparin-independent production of autoantibodies against PF4 [11]. This PF4/polyanion complex resembles the PF4/heparin complex that is responsible for HIT type II and can therefore be found in VITT without any previous heparin exposure [10]. The binding site of anti-PF4 antibodies of VITT patients is restricted to the heparin binding site of PF4 and can actually be inhibited by heparin [13]. Due to the formation of this PF4/polyanion complex [18], thrombocytes are activated and aggregate, leading to thrombophilia with the risk of thromboembolic events and consumption coagulopathy [20]. As opposed to HIT type II, in VITT, bleeding events as in our case have been described frequently [10, 19]. The existence of PF4/vaccine complexes does not necessary result in VITT [20, 21]. Although a positive test with clinical symptoms increases the likelihood of this diagnosis, in some cases of VITT, PF4/vaccine complexes could not even be detected [22–24]. In our case, antibodies against PF4/polyanion complexes could be identified by IgG-specific ELISA, and PF4-related platelet aggregation could be shown by the PIPA test.

### Diagnosis of VITT

Laboratory signs of VITT are significantly elevated D-dimers (up to five times the reference range), thrombocytopenia < 150,000/µl, reduced fibrinogen levels, positive PF4 antibodies in HIT-ELISA, and positive functional platelet activation tests (PAT) such as PIPA and serotonin release assay (SRA) test. Radiographic or ultrasound imaging of an atypical thrombosis can be helpful in the diagnosis of VITT [16, 25]. Atraumatic spontaneous splenic rupture is a rare phenomenon in VITT (with only one other case recorded) [26]. The incidence of atraumatic splenic rupture in the general population is estimated to be around 0.1–0.5% [27]. On suspicion of VITT, it is crucial to implement adequate tests. Studies have revealed that rapid HIT assays such as rapid immunoassays (RIA) or chemiluminescence immunoassays (CLIA) should be avoided as they present poor sensitivity (< 20%) for anti-PF4 antibodies compared with IgG-specific ELISA tests (> 99%) [28, 29]. In our case, the rapid HIT screening test also presented a negative result, in contrast to the positive IgG-specific ELISA. Recommendations for the diagnosis of VITT include PAT such as PF4-SRA, SRA, or HIPA testing with sensitivity > 99% for VITT [24, 25, 29]. Additionally, especially in case of a negative HIPA test, a PIPA test with sensitivity of > 99% should be performed [29]. In our case, a positive PIPA test despite a negative HIPA test confirmed the diagnosis of VITT.

### Treatment of VITT

The pathomechanism of VITT is not fully understood so far, so no direct treatment option is available yet. Management only focuses on treating complications of VITT such as thrombosis and consumption coagulopathy and reducing the amount and effect of circulating PF4 antibodies.

Due to the similarity of VITT to HIT type II, a potential interaction with heparin cannot be excluded. Pending further data, the use of heparin in VITT should be avoided to prevent any possible interaction. Anticoagulation should be performed with nonheparin anticoagulant agents instead, such as danaparoid, argatroban, fondaparinux, or direct oral anticoagulants [30]. Before suspicion of VITT was raised, our patient also received heparin. Whether this had harmful effects remains unclear [3]. After suspicion of VITT, anticoagulation was established with argatroban, a direct thrombin inhibitor with a short plasma half-lifetime, as the patient remained hemodynamically unstable and coagulopathic. The dose of argatroban was monitored by rotation thrombelastometry (ROTEM, Werfen GmbH, Germany) as studies have revealed that aPTT is a suboptimal marker for dosing direct thrombin inhibitors [31–34].

Owing to bleeding complications, our patient required massive transfusion, which in general should be avoided in VITT whenever possible. In particular, platelet transfusions increase the risk of antibody-mediated platelet activation and coagulopathy [30].

As our patient stabilized hemodynamically and his thrombocyte count recovered, anticoagulation could be switched to fondaparinux subcutaneously for the general ward and to an oral anti-Xa inhibitor (apixaban) at discharge to the rehabilitation clinic. Current recommendations for patients suffering from VITT include continuation of therapeutic anticoagulation for at least 3 months [5, 15].

Another treatment option for VITT is the application of IVIG [8, 22, 35], which blocks circulating PF4 antibodies from interacting with the FcγRIIa receptor on the surface of platelets, thereby avoiding platelet activation [3, 5, 11, 36]. However, IVIG therapy itself is associated with a risk of thrombosis in up to 13% when used among patients with autoimmune disorders [37]. Our patient was treated with IVIG (2 × 1 g/kg Octagam). Whether this may have aggravated his thrombosis, requiring radiographically assisted re-thrombectomy, remains unclear.

The use of steroids can be considered in the management of VITT, especially in case of delayed availability or unsuccessful treatment with IVIG and plasma exchange (PLEX) [5]. Corticosteroids potentially increase the thrombocyte count faster than IVIG alone in other settings of autoantibody-mediated thrombocytopenia [38]. Our patient initially received a dose of intravenous corticosteroids (500 mg prednisone). The use of alternative immunosuppressants (rituximab or eculizumab, for example) can be considered in cases of VITT refractory to therapy, but evidence for this treatment is lacking so far [15, 16].

Furthermore plasma exchange (PLEX), preferably against plasma rather than albumin, could represent an alternative option for reducing the amount of circulating PF4/vaccine complexes and correcting hypofibrinogenemia [17]. PLEX was discussed but rejected in our management of VITT as IVIG therapy seemed to be effective.

In our patient, portomesenteric thrombosis was too extensive to be treated conservatively. TIPS insertion was crucial for the successful management of VITT to reduce venous congestion and perform thrombaspiration/thrombolysis via the TIPS catheter [9, 39]. Klinger *et al.* [40] analyzed the safety and benefit of transjugular thrombolysis with or without TIPS in 17 patients with noncirrhotic, nonmalignant portal vein thrombosis. The study could show a high recanalization rate of 94.1% with no major complications. The 1- and 2-year follow-up examination of these patients revealed portal vein patency rates of 88.2%. Transjugular thrombolysis with or without TIPS seems to be a safe and effective measure in acute, noncirrhotic, nonmalignant portal vein thrombosis. This is especially the case in patients presenting with bowel infarction and a low chance of revascularization with systemic anticoagulation, as was the case in our patient. A systematic review of the risks and benefit of thrombolysis and TIPS insertion in VITT-induced portal vein thrombosis is currently lacking, as there is only one other case documented so far, where TIPS insertion and thrombolysis was beneficial in the treatment of VITT [39]. Our patient is the first to be documented with VITT-induced atraumatic splenic rupture requiring splenectomy, TIPS insertion, and thrombolysis for successful management.

## Conclusion

Vaccination with recombinant adenoviral vector vaccine can result in VITT, not rarely with a fatal course. Current treatment options include systemic anticoagulation avoiding heparin and the administration of IVIG, steroids, or other immunosuppressants and PLEX. In rare cases, fulminant and extended portomesenteric thrombosis additionally requires TIPS placement, local thrombaspiration, and lysis for successful management of VITT.

## Data Availability

The datasets used and/or analyzed during the current study are available from the corresponding author on reasonable request. Ethics approval and consent to participate Not applicable.

## Abbreviations

aPTT: Activated prothrombin time
ASS: Acetylsalicylic acid
CLIA: Chemiluminescence immune assays
CT: Computed tomography
DIC: Disseminated intravascular coagulation
DVT: Deep vein thrombosis
ELISA: Enzyme-linked immunosorbent assay
GCS: Glasgow Coma Scale
HIT: Heparin-induced thrombocytopenia
HIPA: Heparin-induced platelet aggregation
HUS: Hemolytic uremic syndrome
ICU: Intensive care unit
IVIG: Intravenous immune globulin
PIPA: PF4-induced platelet aggregation
PAT: Platelet activation test
PF4: Platelet factor 4
PLT: Unit of platelets
RIA: Rapid immune assay
RBC: Unit of red blood cells
SpO_2_: Oxygen saturation
SRA: Serotonin release assay
TIPS: Transjugular portosystemic shunt
tPA: Tissue plasminogen activator
UKK: University Hospital of Cologne
VITT: Vector (vaccine)-induced thrombotic thrombocytopenia

## References

[1] Aleem A, Nadeem AJ. Coronavirus (COVID-19) vaccine-induced immune thrombotic thrombocytopenia (VITT). StatPearls. Treasure Island (FL)2021. 34033367

[2] Mohseni Afshar Z, Babazadeh A, Janbakhsh A, Afsharian M, Saleki K, Barary M, *et al*. Vaccine-induced immune thrombotic thrombocytopenia after vaccination against Covid-19: a clinical dilemma for clinicians and patients. Rev Med Virol. 2021;32:e2273. 10.1002/rmv.2273 PMC8420499 34197678

[3] Alam W. COVID-19 vaccine-induced immune thrombotic thrombocytopenia: a review of the potential mechanisms and proposed management. Sci Prog. 2021;104(2):368504211025927. 10.1177/00368504211025927 PMC10358704 34120531

[4] Schultz NH, Sorvoll IH, Michelsen AE, Munthe LA, Lund-Johansen F, Ahlen MT, *et al*. Thrombosis and thrombocytopenia after ChAdOx1 nCoV-19 vaccination. N Engl J Med. 2021;384(22):2124–30. 10.1056/NEJMoa2104882 PMC8112568 33835768

[5] Asmat H, Fayeye F, Alshakaty H, Patel J. A rare case of COVID-19 vaccine-induced thrombotic thrombocytopaenia (VITT) involving the veno-splanchnic and pulmonary arterial circulation, from a UK district general hospital. BMJ Case Rep. 2021;14(9):e244223. 10.1136/bcr-2021-244223 PMC8451313 34535492

[6] Strobel D, Haberkamp S, Zundler S. Portal vein thrombosis due to vaccine-induced immune thrombotic thrombocytopenia (VITT) after COVID vaccination with ChAdOx1 nCoV-19. Ultraschall Med. 2021;42(5):551–2. 10.1055/a-1579-9303 34598301

[7] Cliff-Patel N, Moncrieff L, Ziauddin V. Renal vein thrombosis and pulmonary embolism secondary to vaccine-induced thrombotic thrombocytopenia (VITT). Eur J Case Rep Intern Med. 2021;8(7): 002692. 10.12890/2021_002692 PMC8276919 34268278

[8] Graf T, Thiele T, Klingebiel R, Greinacher A, Schabitz WR, Greeve I. Immediate high-dose intravenous immunoglobulins followed by direct thrombin-inhibitor treatment is crucial for survival in Sars-Covid-19-adenoviral vector vaccine-induced immune thrombotic thrombocytopenia VITT with cerebral sinus venous and portal vein thrombosis. J Neurol. 2021. 10.1007/s00415-021-10599-2 PMC8140563 34023956

[9] Barral M, Arrive L, El Mouhadi-Barnier S, Cornelis FH. Thromboaspiration and fibrinolysis infusion for portomesenteric thrombosis after AstraZeneca COVID-19 vaccine administration. Intensive Care Med. 2021;47(9):1034–6. 10.1007/s00134-021-06458-3 PMC8206184 34132839

[10] Greinacher A, Thiele T, Warkentin TE, Weisser K, Kyrle PA, Eichinger S. Thrombotic thrombocytopenia after ChAdOx1 nCov-19 vaccination. N Engl J Med. 2021;384(22):2092–101. 10.1056/NEJMoa2104840 PMC8095372 33835769

[11] Pavord S, Scully M, Hunt BJ, Lester W, Bagot C, Craven B, et al. Clinical Features of Vaccine-Induced Immune Thrombocytopenia and Thrombosis. N Engl J Med. 2021. 10.1056/NEJMoa2109908 PMC10662971 34379914

[12] Oldenburg J, Klamroth R, Langer F, Albisetti M, von Auer C, Ay C, *et al*. Diagnosis and management of vaccine-related thrombosis following AstraZeneca COVID-19 vaccination: guidance statement from the GTH. Hamostaseologie. 2021;41(3):184–9. 10.1055/a-1469-7481 33822348

[13] Huynh A, Kelton JG, Arnold DM, Daka M, Nazy I. Antibody epitopes in vaccine-induced immune thrombotic thrombocytopaenia. Nature. 2021;596(7873):565–9. 10.1038/s41586-021-03744-4 34233346

[14] THANZ. Suspected vaccine induced prothrombotic immunthrombocytopenia (VIPIT): THANZ advisory statement for haematologists2021 09.04.2021; 2021.

[15] EHP. Guidance produced from the Expert Haematology panel (EHP) focused on COVID-19 Vaccine induced Thrombosis and Thrombocytopenia (VITT).2021; 2021.

[16] Nazy I, Sachs UJ, Arnold DM, McKenzie SE, Choi P, Althaus K, *et al*. Recommendations for the clinical and laboratory diagnosis of VITT against COVID-19: communication from the ISTH SSC subcommittee on platelet immunology. J Thromb Haemost. 2021;19(6):1585–8. 10.1111/jth.15341 PMC8250233 34018298

[17] Makris M, Pavord S, Lester W, Scully M, Hunt B. Vaccine-induced immune thrombocytopenia and thrombosis (VITT). Res Pract Thromb Haemost. 2021;5(5): e12529. 10.1002/rth2.12529 PMC8178610 34136745

[18] Greinacher A, Selleng K, Palankar R, Wesche J, Handtke S, Wolff M, et al. Insights in ChAdOx1 nCov-19 vaccine-induced immune thrombotic thrombocytopenia (VITT). Blood. 2021. 10.1182/blood.2021013231 PMC8483989 34587242

[19] Rizk JG, Gupta A, Sardar P, Henry BM, Lewin JC, Lippi G, *et al*. Clinical characteristics and pharmacological management of COVID-19 vaccine-induced immune thrombotic thrombocytopenia with cerebral venous sinus thrombosis: a review. JAMA Cardiol. 2021;6:1451. 10.1001/jamacardio.2021.3444 34374713

[20] Greinacher A, Selleng K, Mayerle J, Palankar R, Wesche J, Reiche S, *et al*. Anti-platelet factor 4 antibodies causing VITT do not cross-react with SARS-CoV-2 spike protein. Blood. 2021;138(14):1269–77. 10.1182/blood.2021012938 PMC8294553 34280256

[21] Thiele T, Ulm L, Holtfreter S, Schonborn L, Kuhn SO, Scheer C, *et al*. Frequency of positive anti-PF4/polyanion antibody tests after COVID-19 vaccination with ChAdOx1 nCoV-19 and BNT162b2. Blood. 2021;138(4):299–303. 10.1182/blood.2021012217 PMC8129797 33988688

[22] Chen PW, Tsai ZY, Chao TH, Li YH, Hou CJ, Liu PY. Addressing vaccine-induced immune thrombotic thrombocytopenia (VITT) following COVID-19 vaccination: a mini-review of practical strategies. Acta Cardiol Sin. 2021;37(4):355–64. 10.6515/ACS.202107_37(4).20210628A PMC8261702 34257485

[23] Douxfils J, Favresse J, Dogne JM, Lecompte T, Susen S, Cordonnier C, *et al*. Hypotheses behind the very rare cases of thrombosis with thrombocytopenia syndrome after SARS-CoV-2 vaccination. Thromb Res. 2021;203:163–71. 10.1016/j.thromres.2021.05.010 PMC8123522 34029848

[24] Favaloro EJ. Laboratory testing for suspected COVID-19 vaccine-induced (immune) thrombotic thrombocytopenia. Int J Lab Hematol. 2021;43(4):559–70. 10.1111/ijlh.13629 PMC8444734 34138513

[25] Handtke S, Wolff M, Zaninetti C, Wesche J, Schonborn L, Aurich K, *et al*. A flow cytometric assay to detect platelet-activating antibodies in VITT after ChAdOx1 nCov-19 vaccination. Blood. 2021;137(26):3656–9. 10.1182/blood.2021012064 PMC8105122 33945605

[26] Magee D, Shepherd T, Krisnadi Z, Yau HV, Samuelson S, Radeski D. A unique case of splenic rupture secondary to vaccine-induced immune thrombocytopenia managed with splenic embolization. ANZ J Surg. 2021. 10.1111/ans.17414 34874098

[27] Kocael PC, Simsek O, Bilgin IA, Tutar O, Saribeyoglu K, Pekmezci S, *et al*. Characteristics of patients with spontaneous splenic rupture. Int Surg. 2014;99(6):714–8. 10.9738/INTSURG-D-14-00143.1 PMC4254229 25437576

[28] Platton S, Bartlett A, MacCallum P, Makris M, McDonald V, Singh D, *et al*. Evaluation of laboratory assays for anti-platelet factor 4 antibodies after ChAdOx1 nCOV-19 vaccination. J Thromb Haemost. 2021;19(8):2007–13. 10.1111/jth.15362 PMC8236994 33973336

[29] Warkentin TE. Heparin-induced thrombocytopenia and vaccine-induced immune thrombotic thrombocytopenia antibodies: fraternal-not identical-twins. Thromb Haemost. 2021;121(12):1558–61. 10.1055/a-1585-6264 34384128

[30] Scully M, Singh D, Lown R, Poles A, Solomon T, Levi M, *et al*. Pathologic antibodies to platelet factor 4 after ChAdOx1 nCoV-19 vaccination. N Engl J Med. 2021;384(23):2202–11. 10.1056/NEJMoa2105385 PMC8112532 33861525

[31] Warkentin TE. Anticoagulant failure in coagulopathic patients: PTT confounding and other pitfalls. Expert Opin Drug Saf. 2014;13(1):25–43. 10.1517/14740338.2013.823946 23971903

[32] Beiderlinden M, Werner P, Bahlmann A, Kemper J, Brezina T, Schafer M, *et al*. Monitoring of argatroban and lepirudin anticoagulation in critically ill patients by conventional laboratory parameters and rotational thromboelastometry—a prospectively controlled randomized double-blind clinical trial. BMC Anesthesiol. 2018;18(1):18. 10.1186/s12871-018-0475-y PMC5810183 29426286

[33] Smythe MA, Forsyth LL, Warkentin TE, Smith MD, Sheppard JA, Shannon F. Progressive, fatal thrombosis associated with heparin-induced thrombocytopenia after cardiac surgery despite “Therapeutic” anticoagulation with argatroban: potential role for PTT and ACT confounding. J Cardiothorac Vasc Anesth. 2015;29(5):1319–21. 10.1053/j.jvca.2014.04.029 25169901

[34] Linkins LA, Warkentin TE. Heparin-induced thrombocytopenia: real-world issues. Semin Thromb Hemost. 2011;37(6):653–63. 10.1055/s-0031-1291375 22102268

[35] Bourguignon A, Arnold DM, Warkentin TE, Smith JW, Pannu T, Shrum JM, *et al*. Adjunct immune globulin for vaccine-induced immune thrombotic thrombocytopenia. N Engl J Med. 2021;385(8):720–8. 10.1056/NEJMoa2107051 PMC8362588 34107198

[36] von Hundelshausen P, Lorenz R, Siess W, Weber C. Vaccine-induced immune thrombotic thrombocytopenia (VITT): targeting pathomechanisms with bruton tyrosine kinase inhibitors. Thromb Haemost. 2021;121:1395. 10.1055/a-1481-3039 33851389

[37] Marie I, Maurey G, Herve F, Hellot MF, Levesque H. Intravenous immunoglobulin-associated arterial and venous thrombosis; report of a series and review of the literature. Br J Dermatol. 2006;155(4):714–21. 10.1111/j.1365-2133.2006.07390.x 16965420

[38] Carcao M, Silva M, David M, Klaassen RJ, Steele M, Price V, *et al*. IVMP+IVIG raises platelet counts faster than IVIG alone: results of a randomized, blinded trial in childhood ITP. Blood Adv. 2020;4(7):1492–500. 10.1182/bloodadvances.2019001343 PMC7160294 32282882

[39] Dhoot R, Kansal A, Handran C, Haykal T, Ronald J, Kappus M, *et al*. Thrombocytopenia and splanchnic thrombosis after Ad26.COV2.S vaccination successfully treated with transjugular intrahepatic portosystemic shunting and thrombectomy. Am J Hematol. 2021;96(9):1180–2. 10.1002/ajh.26258 PMC8212098 34057234

[40] Klinger C, Riecken B, Schmidt A, De Gottardi A, Meier B, Bosch J, *et al*. Transjugular local thrombolysis with/without TIPS in patients with acute non-cirrhotic, non-malignant portal vein thrombosis. Dig Liver Dis. 2017;49(12):1345–52. 10.1016/j.dld.2017.05.020 28733177

